# Comparison of non-cryopreserved and cryopreserved autologous hematopoietic stem cell transplantation among patients with myeloma: single-center experience

**DOI:** 10.3389/fonc.2026.1955153

**Published:** 2026-09-24

**Authors:** Lazar Chadievski, Milche Cvetanoski, Sonja Genadieva Stavrikj, Zlate Stojanoski, Marija Popova Labacevska, Dusko Dukovski, Sanja Trajkova, Merve Purde, Martin Stojanoski, Olivera Gjeorgjieva Janev, Arita Fazliu, Aleksandra Pivkova Veljanovska

**Affiliations:** University Clinic for Hematology, Skopje, North Macedonia

**Keywords:** autologous, cryopreservation/, engraftment, hematopoeietic stem cell, myeloma

## Abstract

**Background:**

Non-cryopreserved hematopoietic stem cells (NCP HSCs) may simplify autologous stem cell transplantation (ASCT) by avoiding cryopreservation and dimethyl sulfoxide exposure. We compared the clinical outcomes of NCP HSCs and cryopreserved HSCs (CP HSCs) in patients with multiple myeloma.

**Methods:**

This retrospective, single-center cohort study included 200 consecutive patients with multiple myeloma who underwent ASCT between 2014 and 2024: 147 received NCP HSCs, and 53 received CP HSCs. Preservation practices changed over time, and NCP HSC use increased from 46.6% during 2014–2019 to 94.6% during 2020–2024. The baseline characteristics were not fully balanced: patients receiving NCP HSCs were older (mean age 59.0 vs. 56.5 years), and bortezomib–thalidomide–dexamethasone (VTD) induction was more frequent in the NCP HSC group, whereas cyclophosphamide–thalidomide–dexamethasone (CTD) predominated in the CP HSC group, and the infused CD34^+^ cell dose was higher with NCP HSCs. Sex and myeloma subtype were comparable between the groups, while pre-transplant disease response differed significantly between the cohorts.

**Results:**

The median time to engraftment was 12 days [interquartile range (IQR), 11–13; range, 9–24] with NCP HSCs and 12 days (IQR, 11–14; range, 10–22) with CP HSCs (Mann–Whitney U = 3239, p = 0.062). Median hospitalization was shorter with NCP HSCs [17 days (IQR, 14–20)] than with CP HSCs [23.5 days (IQR, 18–26); p < 0.001]. Transplant-related mortality was 3.4% and 5.7% (p = 0.528), and overall survival was comparable. Mucositis was more frequent in the NCP HSC group, whereas infectious complications did not differ significantly. In multivariable Cox analysis, the stem cell preservation method was not independently associated with progression-free survival (PFS) (adjusted HR, 1.44; 95% CI, 0.76–2.74; p = 0.262). Pre-transplant very good partial response (VGPR) compared with complete response (CR) was associated with a higher progression hazard (adjusted HR, 1.83; 95% CI, 1.10–3.04; p = 0.020), whereas maintenance therapy was associated with a lower progression hazard (adjusted HR, 0.48; 95% CI, 0.26–0.89; p = 0.020).

**Conclusion:**

Compared with CP HSC transplantation, NCP HSC transplantation provided comparable engraftment, transplant-related mortality, and overall survival and was associated with shorter hospitalization. Although PFS differed between the cohorts, multivariable analysis did not identify the stem cell preservation method as an independent predictor of PFS. These findings support NCP HSC transplantation as a feasible alternative when appropriate logistical conditions are available, while emphasizing the contribution of disease- and treatment-related factors to long-term outcomes. NCP HSCs represent an alternative for ASCT in multiple myeloma, although the retrospective design, temporal changes in practice, and baseline differences between the cohorts limit causal interpretation.

## Introduction

Multiple myeloma (MM) is a hematologic malignancy characterized by clonal proliferation of plasma cells in the bone marrow. It accounts for approximately 1%–2% of all malignancies and approximately 10% of all hematological malignancies (1). MM predominantly affects older adults, with a median age at diagnosis of approximately 69 years; only approximately 1% of cases occur in patients younger than 35 years (2). MM is more common in men, who have an approximately 1.5-fold higher risk of developing the disease. MM remains a chronic malignancy that often requires multiple therapeutic approaches throughout the disease course. Advances in modern medicine have provided better overall survival (OS) and progression-free survival (PFS) in treated myeloma patients. The 5-year overall survival has improved from approximately 18% to 64% with the introduction of modern therapies (3). Nevertheless, even in the era of Chimeric Antigen Receptor (CAR) T-cell therapies and bispecific antibodies, autologous stem cell transplantation (ASCT) remains an important component of treatment for eligible patients with multiple myeloma. This is supported by data from the DETERMINATION trial, which showed that upfront autologous hematopoietic stem cell transplantation (HSCT) leads to better disease control and an improved PFS of 67.5 months, compared to 56.2 months in patients treated with chemotherapy alone. In addition, the risk of progression or death was 53% higher in patients without autologous HSCT (4).

Autologous HSCT is basically a four-step process: mobilization of hematopoietic stem cells (HSCs) from the bone marrow into the peripheral blood; apheresis of HSCs via peripheral veins; administration of high-dose chemotherapy as a conditioning regimen, traditionally using melphalan 200 mg/m^2^ for the first transplant procedure and 140 mg/m^2^ for the second transplant procedure or for frail patients; and finally, the reinfusion of HSCs, symptomatic therapy, and supportive care in the early post-transplant period during persistent pancytopenia until engraftment (5). HSC grafts are conventionally cryopreserved using 10% dimethyl sulfoxide (DMSO) as a cryoprotectant. The cells are subsequently stored in liquid nitrogen at approximately −190 °C. However, in some centers, this process demands substantial financial and labor resources. In addition, when a cryopreserved HSCT graft is used for transplantation, it must be thawed to 37 °C and then reinfused into the patient; however, when DMSO enters the bloodstream, it can sometimes cause adverse reactions that affect the cardiovascular, neurological, respiratory, renal, and hepatic systems. Data show that almost one-third of patients are expected to experience DMSO-related reactions. Reports indicate that in patients undergoing autologous transplantation, adverse events occur in 37.8% of infusions. The most frequently reported manifestations were facial flushing (39.4%), nausea and/or vomiting (38.1%), hypoxia requiring supplemental oxygen (29.0%), and chest tightness (16.7%). Although these reactions cannot be attributed exclusively to DMSO, the cryoprotectant is considered an important contributor to the toxicity observed during infusions of cryopreserved grafts. Other factors, including the total infused cell volume, cellular debris, granulocyte content, and infusion rate, may also influence the incidence and severity of adverse events. Overall, severe or life-threatening reactions are uncommon, while mild-to-moderate DMSO-associated toxicity represents a clinically relevant disadvantage of cryopreserved compared with non-cryopreserved autologous hematopoietic stem cell products (6). One option to avoid these kinds of complications is to use non-cryopreserved grafts of HSCs. This approach is supported by data showing that peripheral blood HSCs maintain approximately 85.4% viability for up to 96 h when stored at 1 °C–4 °C after apheresis (7). This fact can form the basis for implementing the use of non-cryopreserved HSCs in autologous HSCT, provided HSCs are properly stored after collection and simply reinfused on day 0 after conditioning with melphalan.

This approach may reduce requirements for storage space, specialized personnel, and substantial financial resources while simplifying the transplantation workflow.

The aim of this study was to evaluate the safety, clinical efficacy, and feasibility of non-cryopreserved hematopoietic stem cells (NCP HSCs) compared with conventional cryopreserved hematopoietic stem cells (CP HSCs) for autologous stem cell transplantation in patients with multiple myeloma, with particular emphasis on hematopoietic recovery, transplant-related complications, hospitalization, and long-term clinical outcomes. We specifically compared hematopoietic engraftment, the incidence of post-transplant complications, the duration of hospitalization, transplant-related mortality (TRM), PFS, and OS between the two transplantation strategies. In addition, we justified avoiding cryopreservation to eliminate DMSO exposure and simplify the stem cell processing and transplantation workflow.

## Methods

We conducted a retrospective cohort study of patients diagnosed with multiple myeloma who underwent autologous HSCT during a period of 10 years (2014–2024) at the University Clinic for Hematology in Skopje. We designed our analysis as a simple comparison of data between two cohorts: patients transplanted with CP HSCs and patients transplanted with NCP HSCs. All transplant procedures were performed in the bone marrow transplant unit at the University Clinic for Hematology in Skopje.

A consecutive sampling approach was used. The sampling was defined and executed as follows: patients with multiple myeloma who underwent ASCT during the study period and fulfilled the eligibility criteria were considered for inclusion. No formal prospective sample-size calculation was performed because of the retrospective nature of the study. The final sample size was therefore determined by the total number of eligible patients treated at our institution during the study period and comprised 200 patients, including 147 receiving NCP HSCs and 53 receiving CP HSCs.

Patients were considered eligible for ASCT according to institutional clinical criteria, including age ≤75 years, Eastern Cooperative Oncology Group (ECOG) performance status of 0–1, absence of clinically significant cardiovascular or respiratory comorbidity that would preclude transplantation, and achievement of at least a very good partial response (VGPR) before transplantation.

All patients were transplanted using the standard conditioning regimen with melphalan 200 mg/m^2^ for the first transplant and 140 mg/m^2^ for the second autologous HSCT or for frail and older patients. All patients were admitted to the bone marrow transplant unit, where the transplantation procedure was performed.

In the early post-transplant period, from aplasia until engraftment, the patients received supportive care with red blood cell transfusions if hemoglobin levels dropped below 80 g/L and platelet transfusions as an absolute indication if the platelets were below 10 × 10^9^/L (exceptions were used in certain circumstances). Standard antibacterial prophylaxis for gram-positive and gram-negative bacteria, and antifungal and antiviral prophylaxes were administered to all patients. Engraftment was defined as a neutrophil count of ≥0.5 × 10^9^/L for three consecutive days, and platelet engraftment was defined as a platelet count of >20 × 10^9^/L without platelet transfusions for three consecutive days (8). The duration of the hospitalization was calculated from the first day of the conditioning until discharge or death.

The post-transplant complications evaluated in this study included mucositis and infectious complications occurring during the early post-transplant period until hematopoietic engraftment and hospital discharge. Infectious complications were defined as clinically and/or microbiologically documented infections requiring antimicrobial treatment. Mucositis was defined as clinically documented oral and/or gastrointestinal mucosal toxicity occurring following conditioning chemotherapy and during the post-transplant aplastic period. Febrile neutropenia was defined as a body temperature above 38 °C when the neutrophil count was <0.5 × 10^9^/L (9).

Regarding the mobilization of peripheral blood HSCs, we used a standard mobilization protocol with Granulocyte Colony-Stimulating Factor (G-CSF) 10–15 μg/kg body weight (BW) for 5 days in one group of patients. For the remaining patients, predominantly those who achieved a VGPR, we performed mobilization using chemotherapy and G-CSF; cyclophosphamide was administered as the standard at a dose of 2 g/m^2^ alongside standard prophylaxis of hemorrhagic cystitis with mesna. Some centers use a higher dose of cyclophosphamide, but we avoid it due to increased toxicity and the lack of significant benefit regarding mobilization effects (10). Plerixafor was not used in any patient.

The apheresis procedure was performed on the Terumo BCT, Inc. (Terumo Blood and Cell Technologies), Lakewood, Colorado, Spectra Optia using an adequate venous access. The minimum required dose of stem cells was defined as 2 × 10^6^/kg BW. For NCP HSC transplantation, the graft was stored at 4 °C. Conditioning was administered the following day, and the stem cells were subsequently infused on day 0. We needed at least 24 h post-melphalan infusion to perform the autologous transplant, although data show that it is safe to do so even sooner, as the half-life of melphalan is much shorter (11). Viability testing was not routinely performed in either the CP HSC or NCP HSC group because it was not part of standard clinical practice and was not available for all patients at our center.

For CP HSCs, stem cell products were cryopreserved using 10% DMSO as a cryoprotectant. The products were initially frozen at −80 °C using a controlled-rate freezing protocol and were subsequently transferred to liquid nitrogen storage at approximately −190 °C. On day 0, the HSC products were thawed at 37 °C and infused after premedication with chloropyramine, methylprednisolone, and acetaminophen.

The primary outcome of the study was hematopoietic recovery following ASCT, assessed by time to neutrophil and platelet engraftment. Secondary outcomes included the duration of hospitalization, TRM, post-transplant complications, PFS, and OS.

Patients were followed up from the date of diagnosis until death, disease progression, last documented clinical assessment, or the end of the study observation period, whichever occurred first. The median follow-up duration for the overall cohort was 3 years (36 months). Progression-free survival was defined as the time from diagnosis to documented disease progression, relapse, or death from any cause, whichever occurred first. Patients without progression or death were censored at the date of their last documented follow-up. Overall survival was defined as the time from diagnosis to death from any cause. Patients who were alive at the end of the observation period were censored at the date of their last documented follow-up. For descriptive comparison between the two transplantation strategies, OS and PFS probabilities were estimated at 12, 24, and 36 months from diagnosis. Time-to-event outcomes over the entire available follow-up period were additionally evaluated using the Kaplan–Meier survival analysis.

Hospitalization duration was calculated from the first day of conditioning therapy until hospital discharge or death. TRM was defined as death in the first 100 days of transplantation, with no evidence of relapse of the disease or its progression (12). During and shortly after the HSC infusion, we monitored for signs, such as nausea/vomiting or cardiac, hepatic, and renal toxicity.

The study was designed, analyzed, and reported in accordance with the Strengthening the Reporting of Observational Studies in Epidemiology (STROBE) recommendations for cohort studies. The STROBE checklist was used to ensure transparent reporting of the study design, setting, participant selection, variables and outcome definitions, potential sources of bias, study size, statistical methods, follow-up, and study limitations.

Given the retrospective and non-randomized design of the study, potential selection bias and confounding were considered during the interpretation of the results. Baseline demographic, disease-related, treatment-related, and transplant characteristics were compared between the NCP HSC and CP HSC cohorts to identify clinically relevant differences between the groups. Particular attention was given to age at transplantation, induction treatment, pre-transplant disease response, and infused CD34^+^ cell dose, as these factors could potentially influence post-transplant and survival outcomes. Because the stem cell preservation strategy was determined via institutional practice rather than random allocation, causal interpretation of between-group differences was avoided.

### Statistical analysis

Data were collected retrospectively from institutional medical records and entered into a specifically designed database for statistical analysis. Continuous variables were summarized using mean and standard deviation (SD) when approximately normally distributed, and median and interquartile range (IQR) when appropriate. Time to engraftment and hospitalization duration were summarized primarily as median (IQR), with ranges reported where relevant, and were compared between the groups using the Mann–Whitney U test. Categorical variables were summarized as absolute frequencies and percentages. Between-group comparisons of continuous variables were performed using parametric or non-parametric statistical tests, as appropriate, according to data distribution. Categorical variables were compared using Pearson’s χ² test or Fisher’s exact test, where appropriate. Effect estimates, including risk ratios and corresponding 95% confidence intervals, were reported when applicable. Survival outcomes were estimated using the Kaplan–Meier method. Differences in PFS and OS between the NCP HSC and CP HSC groups were assessed using the log-rank test. To assess whether the stem cell preservation method was independently associated with PFS, a multivariable Cox proportional hazards regression model was performed. Prespecified covariates included the stem cell preservation method (NCP HSC vs. CP HSC), infused CD34^+^ cell dose, pre-transplant disease response [VGPR vs. complete response (CR)], age at transplantation, and transplant era (2020–2024 vs. 2014–2019). A p-value <0.05 was considered statistically significant. Statistical analyses were performed using GraphPad Prism version 9. The results were presented in tables and graphs and were subsequently analyzed, discussed, and elaborated. During the preparation of the study, and solely for the purpose of achieving the predefined study objectives, additional statistical tests were also applied, where appropriate, to ensure the effective presentation of the analyses, discussion, and conclusions.

## Results

We included 200 patients diagnosed with multiple myeloma and treated at the University Clinic for Hematology in Skopje in the period from 2014 to 2024. All of them underwent autologous HSCT. We divided the patients into two cohorts—patients transplanted with CP HSCs and those transplanted with NCP HSCs—and evaluated the data to show whether the use of NCP HSCs is safe, better, or at least not inferior to autologous transplantation with CP HSCs.

A total of 200 patients with multiple myeloma who underwent autologous stem cell transplantation were included in the study. The cohort comprised 108 men (54.0%) and 92 women (46.0%). Male patients predominated slightly within the cohort. The age at transplantation showed an approximately normal distribution, with a mean of 58.3 ± 7.4 years and a median of 59 years. Most patients were between 50 and 65 years of age at the time of transplantation; within the NCP HSC cohort, the oldest patient was 75 years old, and the youngest was 35 years. Patients receiving cryopreserved stem cells were significantly younger than those receiving fresh stem cells. The median age at transplantation was 58 years in the cryopreserved group compared with 60 years in the fresh stem cell group (Mann–Whitney U test, p = 0.016). Among the 200 patients included in the analysis, IgG multiple myeloma was the most frequent isotype (n = 113, 56.5%), followed by IgA multiple myeloma (n = 40, 20.0%). Bortezomib–thalidomide–dexamethasone (VTD) was the most commonly used induction regimen before autologous stem cell transplantation (n = 116, 58.0%), followed by cyclophosphamide–thalidomide–dexamethasone (CTD) (n = 73, 36.5%). The remaining patients received Bortezomib (Velcade), Cyclophosphamide, and Dexamethasone (VCD), Bortezomib (Velcade), Lenalidomide (Revlimid), and Dexamethasone (VRD), Vincristine, Doxorubicin (Adriamycin), and Dexamethasone (VAD), Bortezomib (Velcade), Melphalan, and Prednison (VMP), or VTD combined with cyclophosphamide. When stratified by ASCT period, NCP HSCs were used in 41 of 88 transplants (46.6%) performed during 2014–2019 and in 106 of 112 (94.6%) during 2020–2024, whereas CP HSC use decreased from 47 of 88 (53.4%) to 6 of 112 (5.4%), demonstrating a significant temporal shift toward the NCP HSC approach (Pearson’s χ² = 58.42, p < 0.001). Mobilization strategies differed significantly between the two groups. All patients in the NCP HSC group were mobilized with G-CSF alone (147/147, 100%), compared with 30 of 53 patients (56.6%) in the CP HSC group. Cyclophosphamide plus G-CSF was used in 23 patients (43.4%) in the CP HSC group and in none of the patients receiving NCP HSCs. Plerixafor was not used in either group. The difference in mobilization strategy between the groups was statistically significant (p < 0.001). The baseline characteristics are summarized in **Table 1**.

**Table 1:** Baseline demographic, disease, treatment, and transplant characteristics according to the stem cell preservation method.

Characteristic	NCP HSCs (n = 147)	CP HSCs (n = 53)	Overall (N = 200)	P-value
Demographic characteristics				
Age at transplantation, years, mean ± SD	59.0 ± 7.3	56.5 ± 7.2	58.3 ± 7.4	
Median age (range), years	60 (35–75)	58 (39–70)	59 (35–75)	0.016
Male sex, n (%)	80 (54.4)	28 (52.8)	108 (54.0)	0.842
Female sex, n (%)	67 (45.6)	25 (47.2)	92 (46.0)	
Disease characteristics				
Multiple myeloma subtype, n (%)				0.98
IgG	83 (56.5)	30 (56.6)	113 (56.5)	
IgA	30 (20.4)	10 (18.9)	40 (20.0)	
Light-chain myeloma	25 (17.0)	9 (17.0)	34 (17.0)	
Other^*^	9 (6.1)	4 (7.5)	13 (6.5)	
Treatment characteristics				
Induction therapy before ASCT, n (%)				<0.001
VTD	95 (64.6)	21 (39.6)	116 (58.0)	
CTD	42 (28.6)	31 (58.5)	73 (36.5)	
Other induction regimens^†^	10 (6.8)	1 (1.9)	11 (5.5)	
Disease response before ASCT, n (%)				0.005
CR	124 (84.4)	35 (66.0)	159 (79.5)	
VGPR	23 (15.6)	18 (34.0)	41 (20.5)	
CD34^+^ cells infused (×10^6^/kg), mean ± SD	5.33 ± 4.46	3.35 ± 1.49	4.81 ± 3.97	<0.001
Transplant characteristics				
Mobilization method, n (%)				<0.001
G-CSF alone	147 (100.0)	30 (56.6)	177 (88.5)	
Cyclophosphamide + G-CSF	0 (0.0)	23 (43.4)	23 (11.5)	
Plerixafor	0 (0.0)	0 (0.0)	0 (0.0)	—
ASCT period, n (%)^‡^				<0.001
2014–2019	41 (27.9)	47 (88.7)	88 (44.0)	
2020–2024	106 (72.1)	6 (11.3)	112 (56.0)	

ASCT, autologous stem cell transplantation; HSCs, hematopoietic stem cells; NCP HSCs, non-cryopreserved hematopoietic stem cells; CP HSCs, cryopreserved hematopoietic stem cells; CR, complete response; VGPR, very good partial response; VTD, bortezomib–thalidomide–dexamethasone; CTD, cyclophosphamide–thalidomide–dexamethasone.

^*^ Other includes plasmacytoma, IgG κ, IgA with extramedullary disease, miscellaneous plasma cell dyscrasias, and patients with unavailable subtype information.

^†^ Other induction regimens include VCD, VRD, VAD, VMP, and VTD combined with cyclophosphamide.

^‡^ ASCT period was assigned according to the transplant episode analyzed within the 2014–2024 study interval. For patients with more than one ASCT, the episode occurring within the study interval was used.

Pre-transplant disease response also differed significantly between the cohorts. Among patients receiving NCP HSCs, 124 of 147 (84.4%) had a CR and 23 (15.6%) had a VGPR. Among patients receiving CP HSCs, 35 of 53 (66.0%) had a CR, and 18 (34.0%) had a VGPR. Overall, 159 patients (79.5%) had a CR, and 41 (20.5%) had a VGPR before ASCT. The difference in response distribution between the two groups was statistically significant (Pearson’s χ² test, p = 0.005) (**Figure 1**).

**Figure 1:**
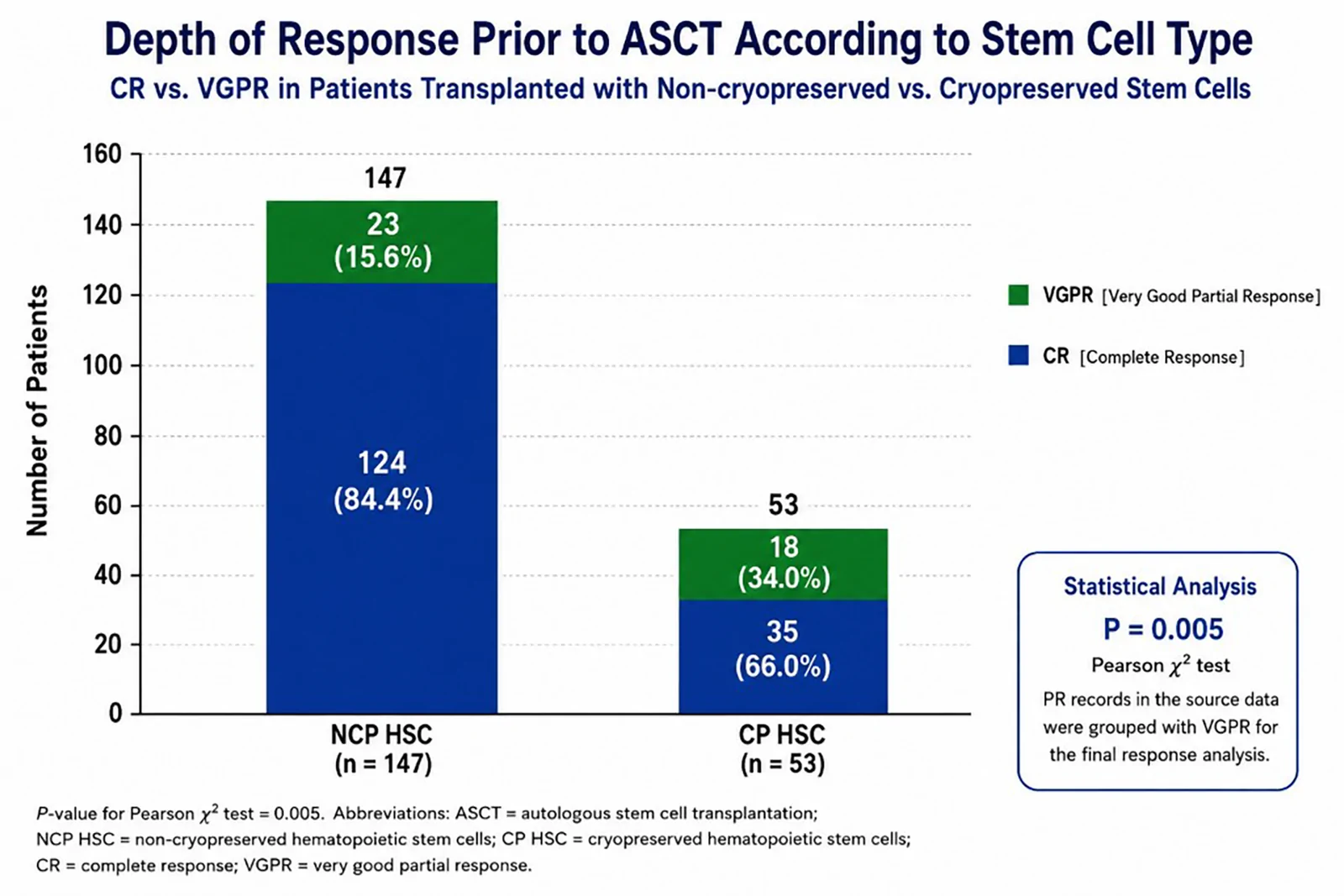
Presentation of depth of response prior to autologous stem cell transplantation (ASCT) according to stem cell type.

In the group of patients who underwent NCP HSCT, the CD34^+^ cell dose was significantly higher than in those transplanted with CP HSCs (mean ± SD: 5.33 ± 4.46 × 10^6^/kg vs. 3.35 ± 1.49 × 10^6^/kg, respectively). The difference between the two groups was statistically significant (Mann–Whitney U test, p < 0.001) (**Figure 2**).

**Figure 2:**
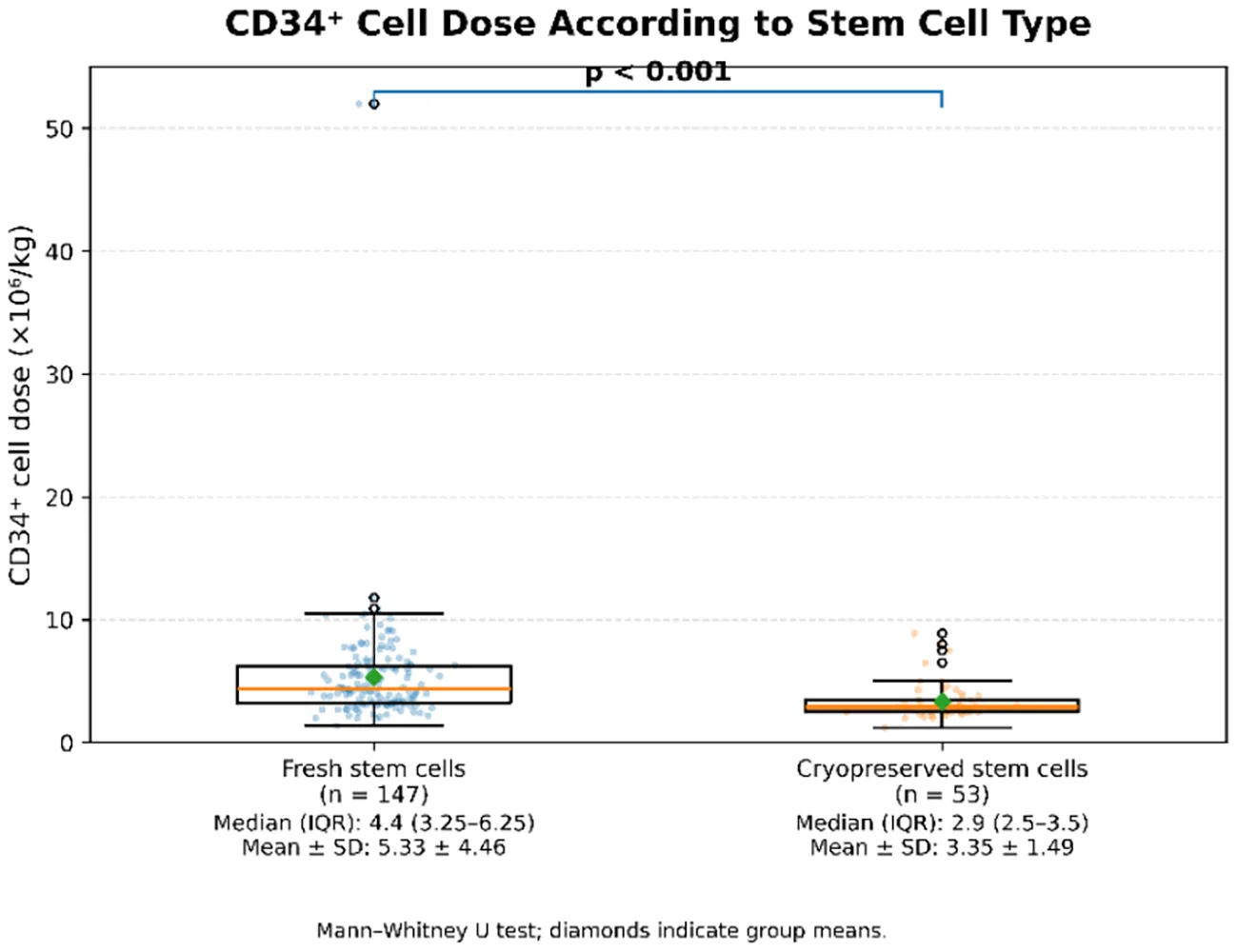
Presentation of CD34^+^ dose according to stem cell type used for autologous HSCT. HSCT, hematopoietic stem cell transplantation.

**Figure 3** compares the incidence of mucositis and infectious complications between the two groups. Mucositis occurred in 45 of 147 patients (30.6%) who received non-cryopreserved hematopoietic stem cells and in 6 of 53 patients (11.3%) who received cryopreserved hematopoietic stem cells. The incidence of mucositis was significantly higher in the NCP HSC group than in the CP HSC group (30.6% vs. 11.3%; p = 0.006). Patients receiving NCP HSCs had an approximately 2.7-fold higher risk of developing mucositis compared with patients receiving CP HSCs (risk ratio, 2.70; 95% confidence interval, 1.22–5.97). Infectious complications were observed in 13 of 147 patients (8.8%) in the NCP HSC group and in 7 of 53 patients (13.2%) in the CP HSC group. Although the incidence was numerically higher among patients receiving cryopreserved stem cells, the difference was not statistically significant (8.8% vs. 13.2%; p = 0.364). The estimated risk ratio was 0.67 (95% confidence interval, 0.28–1.59), indicating that the observed difference may be attributable to sampling variability. The analysis demonstrated a significantly higher incidence of mucositis among patients transplanted with non-cryopreserved hematopoietic stem cells. By contrast, no statistically significant association was identified between the stem cell preservation method and the incidence of infectious complications. These findings suggest that the use of NCP HSCs may be associated with increased mucosal toxicity but does not seem to increase the risk of post-transplant infectious complications. None of the infectious complications was fatal, and all were managed according to the standard local treatment protocol.

**Figure 3:**
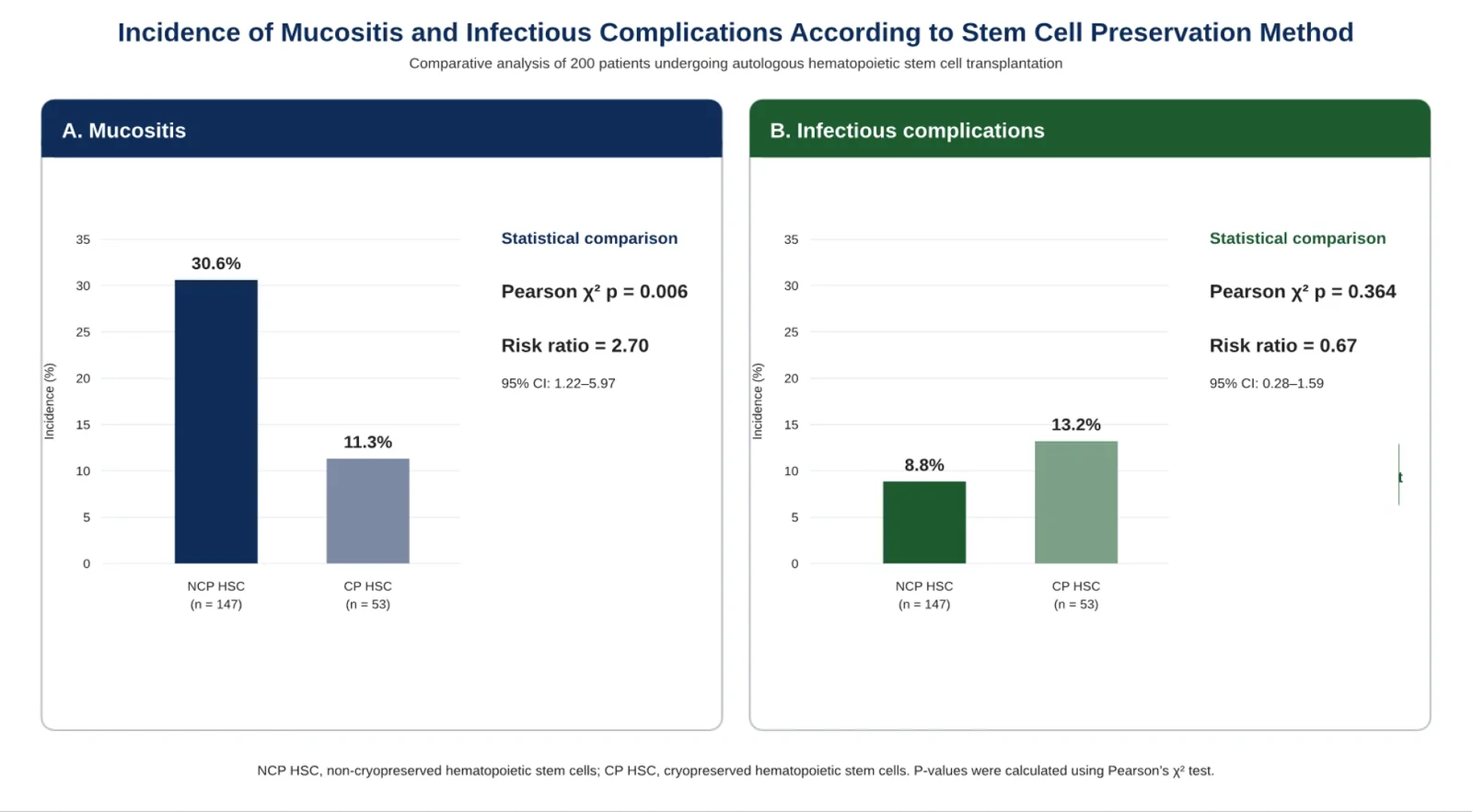
Mucositis and infectious complications.

Time to engraftment was comparable between the two transplantation strategies. The median time to engraftment was 12 days (IQR, 11–13; range, 9–24) in patients receiving NCP HSCs and 12 days (IQR, 11–14; range, 10–22) in patients receiving CP HSCs. There was no statistically significant difference between the groups (Mann–Whitney U = 3239, p = 0.062) (**Figure 4**, **Table 2**).

**Figure 4:**
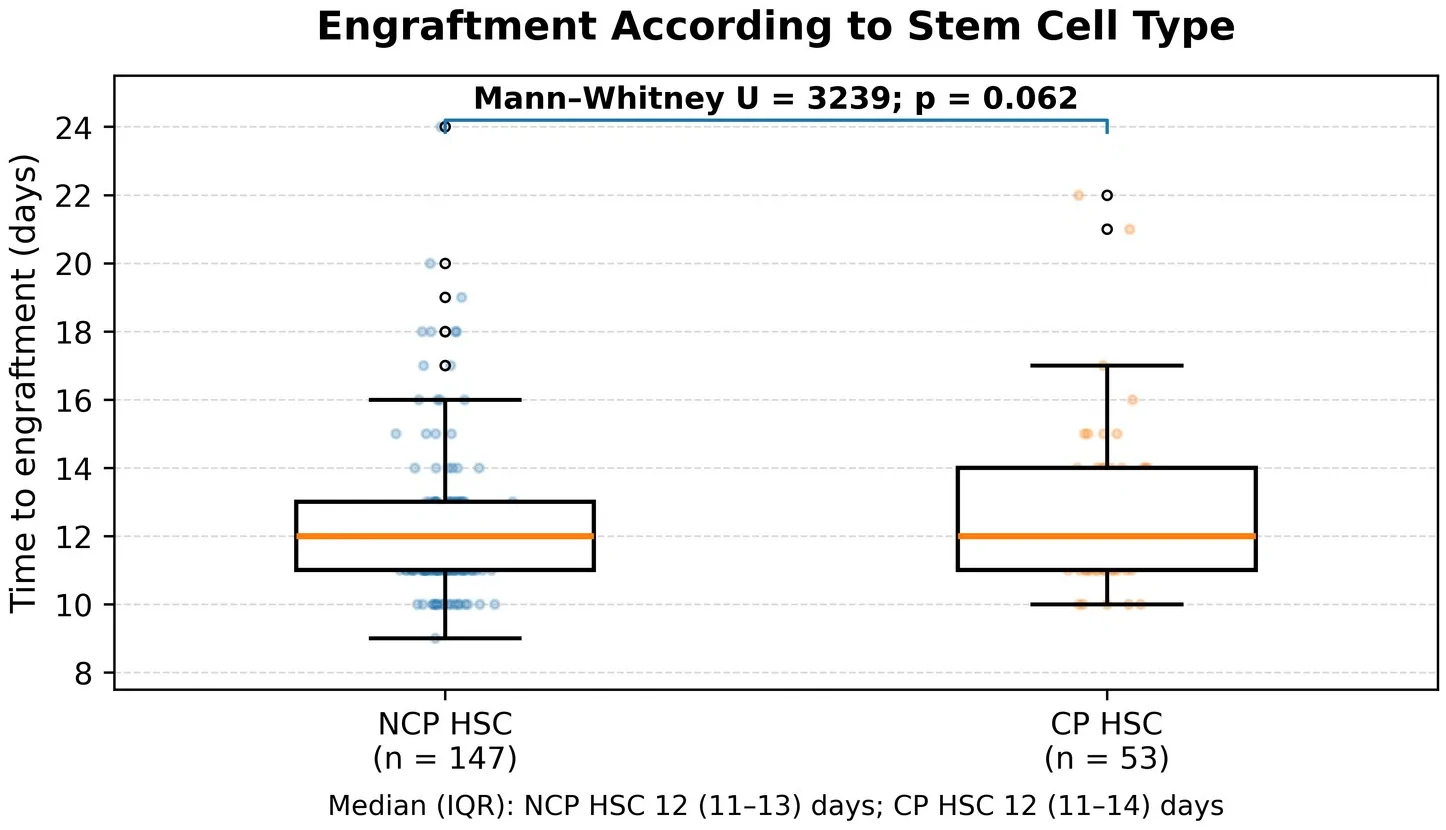
Time to engraftment according to the stem cell preservation method.

**Table 2:** Engraftment characteristics according to the stem cell preservation method.

Engraftment	NCP HSCs (n = 147)	CP HSCs (n = 53)	P-value
Time to engraftment, days, median (IQR)	12 (11–13)	12 (11–14)	0.062
Range, days	9–24	10–22	

Between-group comparison was performed using the Mann–Whitney U test.

NCP HSCs, non-cryopreserved hematopoietic stem cells; CP HSCs, cryopreserved hematopoietic stem cells; IQR, interquartile range.

Hospitalization duration was significantly longer among patients receiving CP HSCs than among those receiving NCP HSCs. Hospitalization data were available for 147 patients in the NCP HSC group and 53 patients in the CP HSC group. Median hospitalization was 17 days (IQR, 14–20; range, 10–37) in those transplanted with NCP HSCs and 23.5 days (IQR, 18–26; range, 14–39) in those transplanted with CP HSCs. The difference was statistically significant (Mann–Whitney U = 1805.5, p < 0.001) (**Table 3**).

**Table 3:** Hospitalization duration according to the stem cell preservation method.

Stem cell product	Available data, n	Median (IQR), days	Range, days	P-value
NCP HSCs	147	17 (14–20)	10–37	<0.001
CP HSCs	53	23.5 (18–26)	14–39	

Between-group comparison was performed using the Mann–Whitney U test.

NCP HSCs, non-cryopreserved hematopoietic stem cells; CP HSCs, cryopreserved hematopoietic stem cells; IQR, interquartile range.

Patients who received CP HSCs had a significantly longer hospitalization duration than those who received NCP HSCs, with a 6.5-day difference between group medians.

The Kaplan–Meier analysis demonstrated comparable overall survival between patients transplanted with NCP HSCs and CP HSCs. The estimated OS rates at 1, 2, and 3 years were 96.6%, 89.1%, and 81.6% for the NCP HSC group and 96.2%, 90.4%, and 80.7% for the CP HSC group, respectively. No statistically significant difference was observed between the groups (log-rank χ² = 0.44, p = 0.505).

The estimated PFS rates at 1, 2, and 3 years were 94.4%, 81.1%, and 73.4% in the NCP HSC group compared with 92.3%, 84.6%, and 63.0% in the CP HSC group. The Kaplan–Meier analysis demonstrated a statistically significant difference in PFS between the two cohorts (log-rank p = 0.0329), with higher estimated PFS in the NCP HSC cohort. Given the retrospective design, baseline differences, and temporal changes in treatment practice, this association should not be interpreted as a causal effect of the preservation method (**Figure 5**).

**Figure 5:**
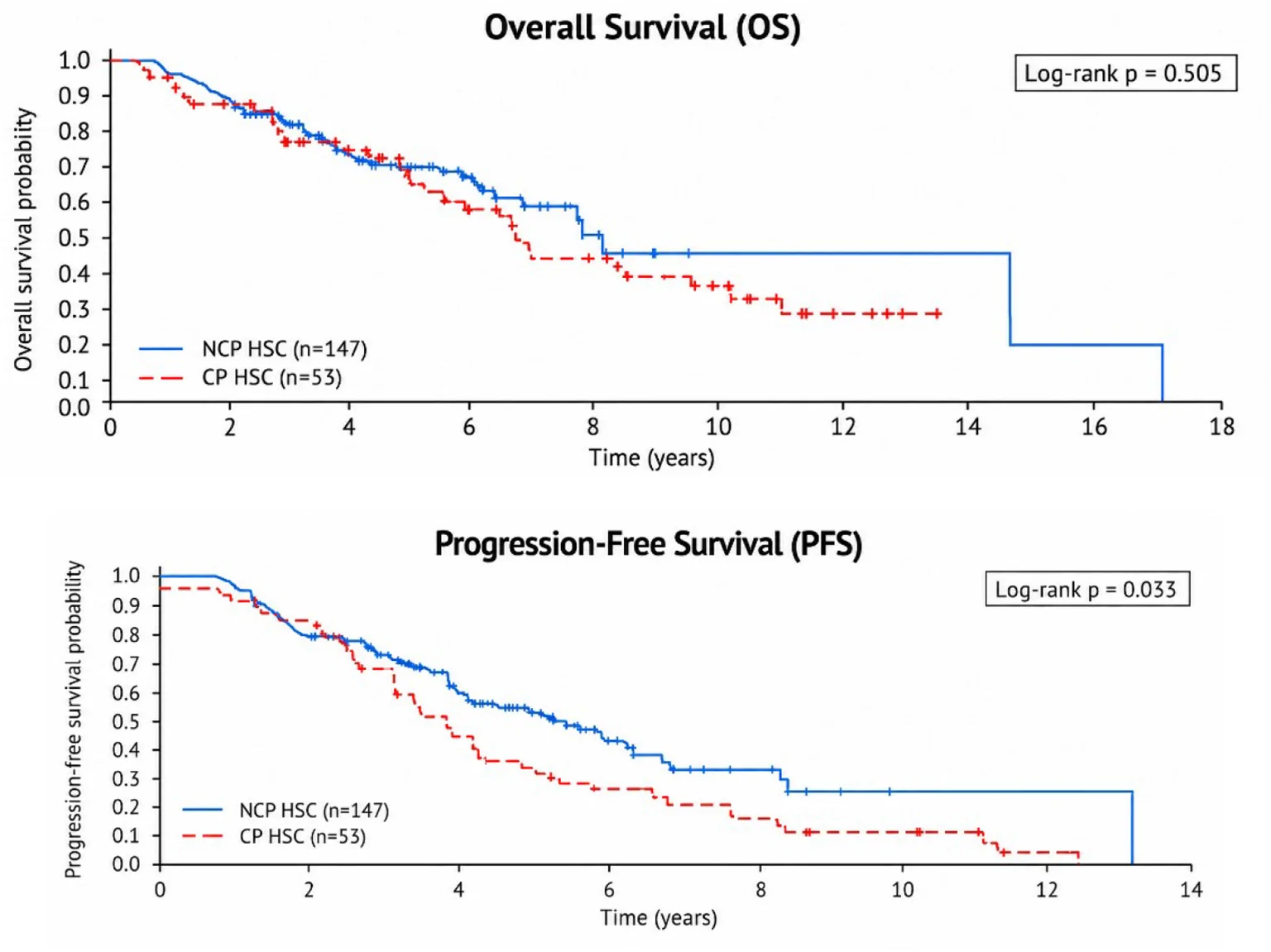
OS and PFS in patients transplanted with CP and NCP HSCs. OS, overall survival; PFS, progression-free survival; CP, cryopreserved; NCP HSCs, non-cryopreserved hematopoietic stem cells.

We performed an analysis of TRM in our group of patients. Among 200 patients included in the analysis, TRM occurred in 5 of 147 patients (3.4%) receiving NCP HSCs and in 3 of 53 patients (5.7%) receiving CP HSCs. Comparison of TRM rates demonstrated no statistically significant difference between the two groups (p = 0.528). Although the TRM rate was numerically higher in the CP HSC group, the observed difference did not reach statistical significance (**Figure 6**).

**Figure 6:**
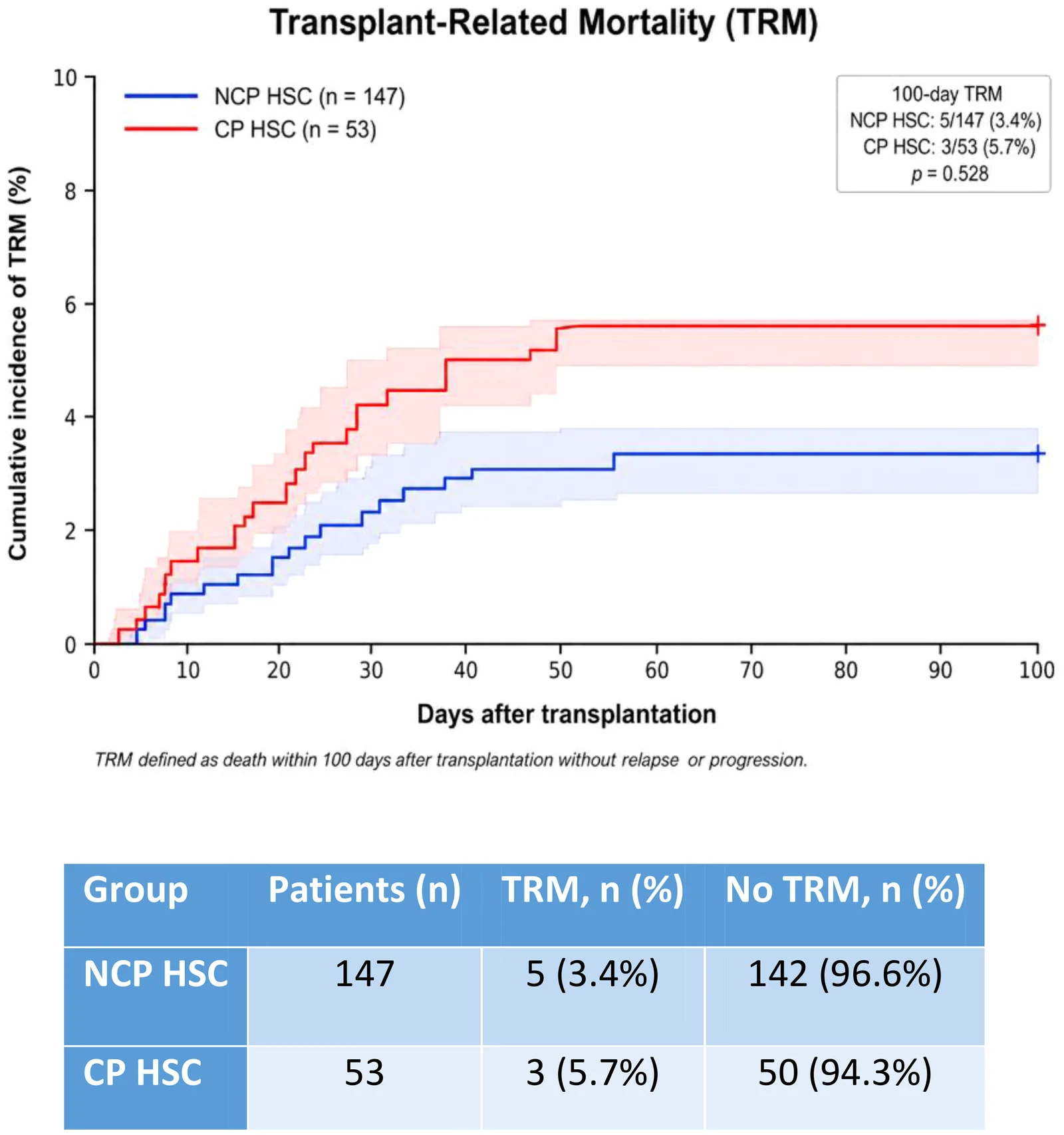
TRM in patients transplanted with NCP HSCs and CP HSCs. TRM, transplant-related mortality; NCP HSCs, non-cryopreserved hematopoietic stem cells; CP, cryopreserved.

Early post-transplant clinical outcomes and toxicities according to stem cell preservation strategy are summarized in **Table 4**. Time to engraftment and infectious complication rates were comparable between the two groups, whereas hospitalization was significantly shorter in the NCP HSC group. Mucositis occurred more frequently among patients receiving NCP HSCs, while TRM did not differ significantly between the two transplantation strategies.

**Table 4:** Clinical outcomes and early post-transplant toxicities according to the stem cell preservation method.

Outcome	NCP HSCs (n = 147)	CP HSCs (n = 53)	P-value
TRM, n (%)			0.528
Yes	5 (3.4%)	3 (5.7%)	
No	142 (96.6%)	50 (94.3%)	
Hospitalization, days			<0.001
Available data, n	147	53	
Median (IQR)	17 (14–20)	23.5 (18–26)	
Range	10–37	14–39	
Mucositis, n (%)	45 (30.6%)	6 (11.3%)	0.006
Infectious complications, n (%)	13 (8.8%)	7 (13.2%)	0.364

NCP HSCs, non-cryopreserved hematopoietic stem cells; CP HSCs, cryopreserved hematopoietic stem cells; IQR, interquartile range; TRM, transplant-related mortality.

To assess whether the stem cell preservation method was independently associated with PFS, a multivariable Cox proportional hazards regression analysis was performed. The complete-case model was adjusted for the stem cell preservation method, infused CD34^+^ cell dose, pre-transplant disease response, age, transplant era, induction regimen, and maintenance therapy. After adjustment, NCP HSC transplantation was not independently associated with PFS compared with CP HSC transplantation (adjusted HR, 1.44; 95% CI, 0.76–2.74; p = 0.262). By contrast, patients with a VGPR before ASCT had a significantly higher risk of progression or death compared with those with a CR (adjusted HR, 1.83; 95% CI, 1.10–3.04; p = 0.020), while maintenance therapy was associated with a lower risk of progression or death (adjusted HR, 0.48; 95% CI, 0.26–0.89; p = 0.020). CD34^+^ cell dose, age, transplant era, and induction regimen were not independently associated with PFS. These findings indicate that the significant difference observed in the unadjusted Kaplan–Meier PFS analysis was not independently explained by the stem cell preservation method after adjustment for relevant clinical and treatment-related factors (**Table 5**).

**Table 5:** Multivariable Cox analysis for progression-free survival (PFS).

Variable	Reference	Adjusted HR	95% CI	P-value
NCP HSCs	CP HSCs	1.44	0.76–2.74	0.262
CD34^+^ dose, per 1 × 10^6^/kg	—	0.97	0.84–1.11	0.619
VGPR	CR	1.83	1.10–3.04	0.020
Age, per year	—	1.00	0.97–1.03	0.823
ASCT era 2020–2024	2014–2019	0.62	0.28–1.39	0.243
CTD induction	VTD	0.87	0.40–1.86	0.712
Other induction regimen	VTD	0.50	0.14–1.82	0.293
Maintenance therapy: yes	No maintenance	0.48	0.26–0.89	0.020

NCP HSCs, non-cryopreserved hematopoietic stem cells; CP HSCs, cryopreserved hematopoietic stem cells; VGPR, very good partial response; CR, complete response; ASCT, autologous stem cell transplantation; CTD, cyclophosphamide–thalidomide–dexamethasone; VTD, bortezomib–thalidomide–dexamethasone.

## Discussion

ASCT remains an important component of first-line therapy for transplant-eligible patients with multiple myeloma, even in the era of proteasome inhibitors, immunomodulatory agents, monoclonal antibodies, bispecific antibodies, and CAR T-cell therapies. Accordingly, optimization of stem cell processing and effective transplant procedure is clinically relevant, particularly when simplification of the procedure can be achieved without compromising safety or efficacy. Previous clinical series, comparative studies, and recent international practice considerations have shown that non-cryopreserved peripheral blood stem cells stored under controlled refrigeration can be used successfully for autologous transplantation in multiple myeloma (13–20). The findings from this single-center experience are consistent with the published evidence and support NCP HSC transplantation as an alternative to conventional cryopreserved grafts. A long-term, single-center experience has also supported the feasibility of this approach. Bekadja et al. reported a 10-year experience with non-cryopreserved autologous transplantation involving 420 patients with multiple myeloma, with graft viabilities exceeding 90%, consistent engraftment, and acceptable early transplant mortality (22).

In our cohort, preservation strategy did not seem to adversely affect overall survival, transplant-related mortality, or hematopoietic recovery. These observations are consistent with comparative studies and contemporary pooled evidence reporting reliable engraftment and acceptable early transplant outcomes with non-cryopreserved grafts adequately stored without compromising their viability (14). The Worldwide Network for Blood and Marrow Transplantation has likewise concluded that non-cryopreserved autologous peripheral blood stem cell transplantation can be a feasible and safe approach when collection, storage, conditioning, and reinfusion are carefully coordinated (18). Taken together, the available evidence suggests that short-term refrigerated storage can preserve sufficient graft function for dependable hematopoietic reconstitution (7, 15). Our observations are consistent with previous comparative studies and contemporary evidence demonstrating reliable engraftment and acceptable early transplant outcomes with non-cryopreserved grafts. Importantly, a recent large matched analysis from the Chronic Malignancies Working Party of the European Society for Blood and Marrow Transplantation (EBMT) compared 367 patients with multiple myeloma receiving non-cryopreserved peripheral blood stems (PBSCs) with 1,412 patients receiving cryopreserved PBSCs. The median neutrophil and platelet engraftment times were comparable between the two strategies, and no significant difference in day +100 non-relapse mortality was observed (21).

The more favorable progression-free survival observed in the NCP HSC cohort should be interpreted cautiously and should not be attributed directly to the preservation method. Interestingly, the recent EBMT analysis also reported significantly longer PFS in the non-cryopreserved cohort, while overall survival remained comparable between the groups (21). Our study is a retrospective, non-randomized comparison spanning a long treatment period, and the two cohorts differed in the distribution of induction regimens before transplantation. Because treatment exposure and treatment era may influence post-transplant disease control, residual confounding is a plausible explanation for the observed difference. The established viability and clinical performance of peripheral blood stem cells during short-term refrigerated storage support the biological feasibility of NCP HSC transplantation, but they do not by themselves establish a survival advantage over cryopreservation (7, 13–20). The observed PFS difference may be partly explained by differences in the treatment regimen mostly used in the group of patients transplanted with CP HSCs as an induction therapy. It was a historical CTD regimen, and it is highly inferior to the VTD regimen, which was the standard of care for the majority of patients with multiple myeloma who were transplanted with NCP HSCs. In addition, the present multivariable analysis substantially changes the interpretation of this observation. After adjustment for CD34^+^ cell dose, pre-transplant response, age, transplant era, induction regimen, and maintenance therapy, the stem cell preservation method was no longer independently associated with PFS. Therefore, the significant unadjusted Kaplan–Meier difference should not be interpreted as evidence that the use of NCP HSCs itself improves disease control.

Engraftment kinetics were broadly comparable between strategies, and the preservation method was not associated with an evident excess of infectious complications. Similar hematopoietic recovery with non-cryopreserved grafts has been reported by several groups, including recent comparative studies (17–19). Contemporary data also suggest that avoiding cryopreservation may reduce some early infusion-related toxicities while maintaining effective engraftment (19, 20). The higher frequency of mucositis observed in the NCP HSC cohort is therefore unlikely to be explained solely by impaired graft function and may instead reflect differences in patient characteristics, treatment era, supportive-care practices, conditioning-related toxicity, or other unmeasured factors. This finding should be considered exploratory and warrants confirmation in cohorts with more detailed toxicity grading and adjustment for potential confounders.

A clinically relevant advantage of the NCP HSC strategy in our series was shorter hospitalization. Previous and recent comparative studies have likewise reported shorter inpatient stay or reduced early resource utilization when cryopreservation is omitted (13–15, 19, 20). In practical terms, a shorter inpatient course can improve bed availability and reduce the intensity of supportive-care resource use, although the magnitude of any economic benefit will depend on local staffing, laboratory organization, and hospitalization costs. A formal health-economic analysis was not performed in the present study, and cost savings should therefore be regarded as a plausible operational benefit rather than a directly measured outcome.

Conventional cryopreservation requires controlled freezing, storage at very low temperatures, quality-control procedures, thawing, and handling of a DMSO-containing product. DMSO exposure is associated with infusion-related adverse effects, which are most commonly mild to moderate but can potentially involve the cardiovascular, respiratory, neurological, renal, and hepatic systems (6). Recent comparative studies have also reported a greater burden of infusion-related reactions or early transplant-related toxicity with cryopreserved grafts (19, 20). Non-cryopreserved transplantation avoids DMSO exposure and eliminates several laboratory steps associated with freezing, storage, and thawing (13–20). These features may be particularly relevant in centers with limited infrastructure, as emphasized in recent Worldwide Network for Blood and Marrow Transplantation (WBMT) practice considerations, and may also simplify workflow in established high-volume transplant programs (18).

The higher CD34^+^ cell dose observed in the NCP HSC cohort should not be interpreted as an effect of the preservation strategy itself, as cell yield is primarily influenced by mobilization, collection, patient characteristics, and clinical practice. Adequate CD34^+^ cell collection remains central to successful autologous transplantation, and both preservation approaches can provide grafts capable of prompt hematopoietic recovery when accepted collection thresholds are achieved (8, 10, 19, 20). In this context, the comparable engraftment observed in our study is more informative regarding functional graft adequacy than the between-group difference in collected cell dose.

The study has several limitations. Its retrospective, single-center design, unequal cohort sizes, differences in induction treatment between the groups, and the long inclusion period create the potential for selection bias, temporal bias, and residual confounding. Post-storage cell viability and functional assays were not routinely available, preventing direct correlation between storage conditions and graft quality. In addition, formal cost-effectiveness analysis and standardized prospective grading of infusion-related toxicities were not performed. These limitations preclude causal conclusions regarding preservation strategy, particularly for long-term outcomes. Data on Revised International Staging System (R-ISS) risk stratification were not consistently available across the entire study period and therefore could not be incorporated into the comparative or multivariable analyses. Nevertheless, the consecutive real-world experience over the study period, together with findings that are broadly consistent with published studies and recent international evidence on non-cryopreserved autologous transplantation (13–20), supports further evaluation and structured implementation of this approach in appropriately organized transplant programs.

## Conclusion

NCP HSCs represent a feasible alternative to conventional cryopreserved transplantation and may offer resource-utilization and logistical advantages when transplantation can be appropriately coordinated. The absence of significant differences in overall survival, TRM, engraftment kinetics, and infectious complications suggests that NCP HSC transplantation does not adversely affect these clinical outcomes. Moreover, the markedly shorter hospitalization and the elimination of cryopreservation-related infrastructure, laboratory procedures, DMSO exposure, and associated costs may simplify stem cell processing and reduce requirements for specialized infrastructure. However, formal cost-effectiveness was not evaluated in this study. NCP HSC transplantation may represent a practical strategy for simplifying transplant workflow while maintaining clinically acceptable outcomes. Our findings support NCP HSC transplantation as a feasible alternative when appropriate logistical conditions are available.

## Data Availability

The data analyzed in this study is subject to the following licenses/restrictions: it is a database from the archive of the university clinic for hematology in Skopje, Republic of North Macedonia (retrospective analysis). Requests to access these datasets should be directed to laze_cad@yahoo.com.
